# Pro-inflammatory and anti-inflammatory cytokine expression in post-treatment apical periodontitis

**DOI:** 10.1590/1678-7757-2017-0455

**Published:** 2018-05-22

**Authors:** Nilton Dessaune, Mariana Teixeira Maneschy Porpino, Henrique dos Santos Antunes, Renata Costa Val Rodrigues, Alejandro Ron Perez, Fábio Ramôa Pires, José Freitas Siqueira, Luciana Armada

**Affiliations:** 1 Universidade Estácio de Sá, Faculdade de Odontologia, Departamento de Endodontia, Rio de Janeiro, RJ, Brasil.; 2 Universidade Veiga de Almeida, Faculdade de Odontologia, Departamento de Endodontia, Rio de Janeiro, RJ, Brasil.

**Keywords:** Periapical periodontitis, Periapical granuloma, Radicular cyst, Cytokines

## Abstract

**Objective:**

This study evaluated the expression of pro-inflammatory (IL-1β, IL-6, IFN-γ and TNF-α) and anti-inflammatory (IL-4 and TGF-β) cytokines in apical periodontitis lesions. Correlations between these cytokines and clinical and cone-beam computed tomographic (CBCT) data were also assessed.

**Material and Methods:**

Apical periodontitis lesions’ data were obtained from 27 patients subjected to periradicular surgery. Specimens were processed for histopathologic and immunohistochemical analysis. Sections were evaluated according to the amount of positive staining for each antibody. Expression levels of the target mediators were compared with clinical and CBCT data.

**Results:**

Twenty lesions were diagnosed as granuloma and 7 as cyst. In granulomas, IL-4 expression was significantly higher than IL-6 (p=0.001) and TNF-α (p=0.001). There was a significant relationship between high levels of TNF-α and lesions <5 mm (p=0.017). In cysts, IL-6 expression was significant lower than IL-4 (p=0.001) and IFN-γ (p=0.004). There was a significant relationship between high levels of TGF-β and endodontic treatment performed ≤4 years before (p=0.045). In general, IL-4 was the most expressed mediator in both cysts and granulomas.

**Conclusions:**

There was a balance between the expression of pro-inflammatory and anti-inflammatory cytokines associated with the chronic periradicular inflammatory process. TNF-α and TGF-β were related to some clinical and CBCT data.

## Introduction

Apical periodontitis (AP) consists of a host tissue inflammatory response to bacterial infection of the root canal and serves the purpose of curbing the infection advance towards the bone and other body parts^23^. Bacteria cause direct damage to the periradicular tissues by the action of exotoxins, enzymes and metabolites^24^, but their main effects in the pathogenesis of AP is highly likely to be related to cellular structural components and other released factors that cause indirect tissue damage by stimulating and modulating the host inflammatory response^22^.

Bone destruction is a common feature of chronic AP and results from the release of mediators that induce osteoclast formation and activation during inflammation^13^. The bone resorption process in the periradicular region is modulated by pro-inflammatory cytokines, such as interleukin (IL)-1β, IL-6, interferon (IFN)-γ and tumor necrosis factor (TNF)-α, and anti-inflammatory cytokines, such as transforming growth factor (TGF)-β and IL-4. Most of these cytokines are upregulated in response to bacterial infection and the balance between pro- and anti-inflammatory cytokines controls the extent and outcome of the host immune response to antigen stimulation during the chronic inflammatory process. Actually, pro-inflammatory mechanisms must be properly controlled to prevent excessive tissue destruction^8^ ^,^ ^15^. Studies have indicated that a cytokine network is activated in the periradicular tissues in response to bacterial infection, in which pro-inflammatory pathways predominate during active bone destruction, while anti-inflammatory pathways prevail in phases of lesion stabilization^22^. Numerous studies have detected pro-inflammatory and anti-inflammatory cytokines in AP lesions^1^ ^,^ ^7^ ^-^ ^11^ ^,^ ^13^ ^-^ ^15^, suggesting that destructive and protective immune mechanisms must coexist in the inflammatory periradicular response. There is a scarcity of information correlating the expression of pro- and anti-inflammatory cytokines with clinical and imaging characteristics of AP. Therefore, the purpose of this study was to evaluate the expression of pro-inflammatory (IL-1β, IL-6, IFN-γ and TNF-α) and anti-inflammatory (IL-4 and TGF-β) cytokines in human AP lesions (granulomas and cysts), and evaluate any relationship between their expression levels and clinical and cone-beam computed tomography (CBCT) data.

## Material and methods

The research project was approved by the Institutional Research Ethics Committee (CAAE: 47669715.2.0000.5284). We have read the Helsinki Declaration and followed its guidelines in this investigation. Specimens consisted of AP lesions obtained by periradicular surgery, all performed by the same endodontist in a private practice (H.S.A.). Through anamnesis and clinical examination, information was gathered with reference to demographic characteristics (age and gender), symptoms, presence of sinus tracts, and the time elapsed since the root canal treatment before surgery. CBCT scans were available for all cases as requested for surgery planning and were used to determine the location and size of the AP lesion. The largest diameter of the lesions in the 3D CBCT analysis was classified as small when it was <5 mm or large when it was ≥5 mm. Specimens were excluded from this study when they were: of insufficient size for histologic processing, from patients with immunosuppressive diseases (such as diabetes mellitus, acquired immunodeficiency syndrome or auto-immune diseases), and from individuals who made use of analgesic, anti-inflammatory and/or antibiotic agents one month before surgery.

The specimens were obtained in one piece by curettage during surgery. They were fixed in 10% formalin medium and processed for histological analysis. The histopathologic diagnosis was made by two experienced evaluators by slides stained with hematoxylin and eosin. Lesions that presented a cavity partially or totally lined by epithelium were classified as cysts. Therefore, 7 lesions were cysts and the other 20 were granulomas.

Histological sections were mounted on silanized slides for performing the immunohistochemical reactions according to a previously described protocol^3^ ^,^ ^7^. The following antibodies were used: primary antibodies for IL-1β (1:100, rabbit, sc-7884), IL-6 (1:500, mouse, sc-130326), IFN-γ (1:200, rabbit, sc-8308), TGF-β (1:100, rabbit, sc-146)) and TNF-α (1:50, mouse, sc-130349) from Santa Cruz Biotechnology (Dallas, TX, USA); and the primary antibody for IL-4 (1:1000, mouse, 25463) from RD Systems (Minneapolis, MN, USA). The LSAB + HRP system (Dako K0690, DAKO North America, Carpinteria, CA, USA) was used as secondary antibody. Liquid DAB (Liquid DAB + Substrate Chromogen System - Dako K3468, DAKO North America, Carpinteria, CA, USA) was used to stain the areas of cytokine expression. Positive and negative controls were performed for each antibody used, following the manufacturers’ instructions.

Images were independently analyzed by two blinded evaluators, being previously calibrated with an optical microscope (Leica DM500, Heerbrugg, Sweden). Each specimen was divided into 5 fields and the epithelium (cysts) and connective tissue were analyzed under high-power view (400x); the expression values were obtained from the number of positive cells in each field. Each are observed was categorized according to the percentage of positive staining and the following scores were assigned: 0, negative/focal, if there were no positive cells or <5% of the cells were positively stained; 1, weak to moderate, if >5% to 50% of the cells were positively stained; and 2, strong, if >50% of the cells were positive. The mean score for the whole specimen was calculated and the expression of the target cytokines was ranked as negative/focal (final mean ranging from 0 to 0.5), weak to moderate (ranging from 0.6 to 1.2), and strong (ranging from 1.3 to 2.0). At the end of the evaluation, results were compared. Discrepant cases were resolved by a third and more experienced evaluator.

Comparative analysis of the data obtained was performed using the SPSS program (Statistical Program for Social Sciences, version 2.1, IBM, São Paulo, SP, Brazil). For associations between the cytokine expression levels and clinical/CBCT data, the Mann-Whitney non-parametric test was used. Statistical significance was set at p<0.05. For comparison of expression levels between the target cytokines, the Friedman and Wilcoxon multiple comparison tests with Bonferroni correction were carried out. Statistical significance was set at p<0.008.

## Results

**Figure 1:**
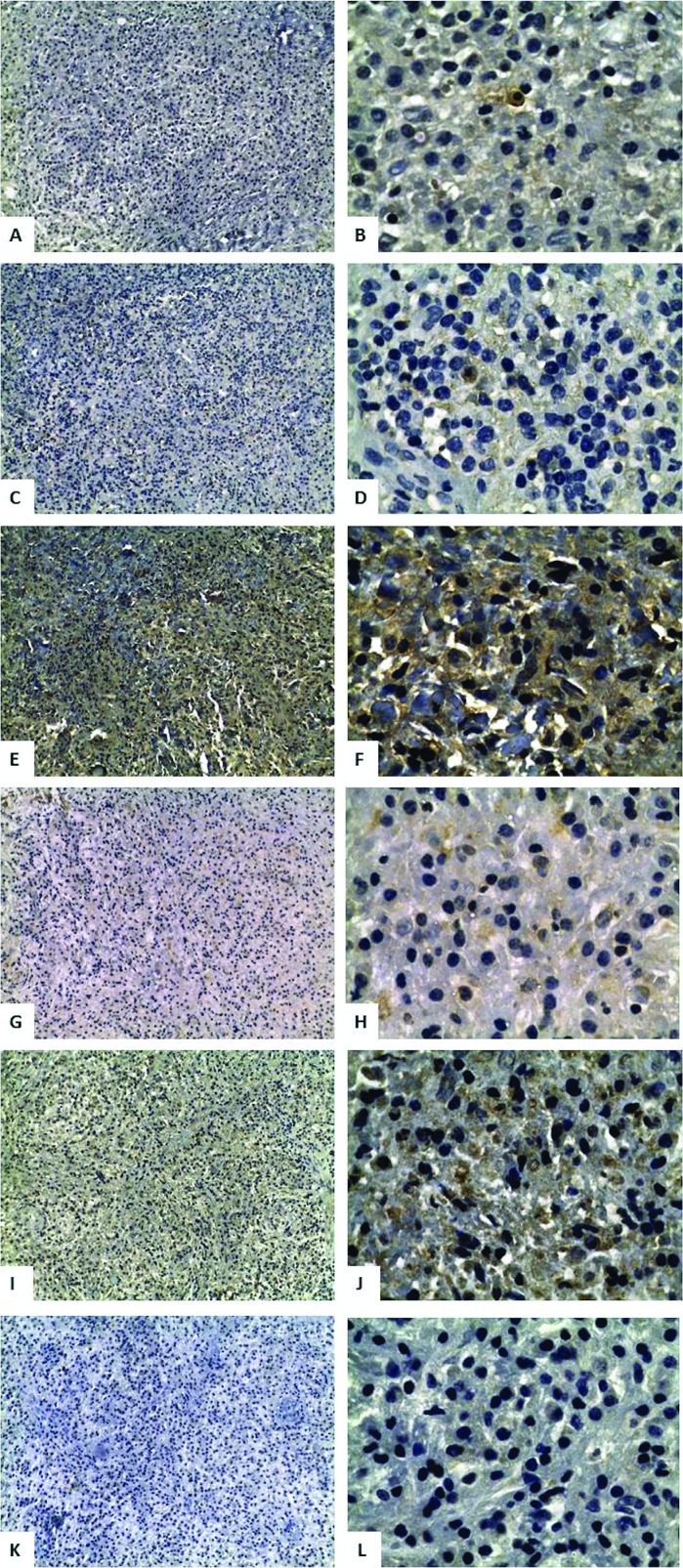
Apical periodontitis specimens with positive staining for pro-inflammatory and anti-inflammatory cytokines. A, IFN-γ (200x); B, IFN-γ (1000x); C, IL-1β (200x); D, IL-1β (1000x); E, IL-4 (200x); F, IL-4 (1000x); G, IL-6 (200x); H, IL-6 (1000x); I, TGF-β (200x); J, TGF-β (1000x); K, TNF-α (200x); L, TNF-α (1000x)

Demographic, clinical and CBCT data are displayed in Table 1. Evaluation of cytokine expression in AP lesions revealed that focal expression was higher for all cytokines, except for IL-4 (Table 2, Figure 1). IL-4 was the most intensely expressed cytokine in both cysts and granulomas. No specimen exhibited negative staining for the target cytokines.

**Table 1:** Demographic, clinical, and cone-beam computed tomographic data

Patient Data		Apical periodontitis lesion/Teeth data	
**Age (years)**		**Tooth location**	
Mean (SD)	51.7±14.3	Maxilla	18 (66.6%)
Range	32-78	Mandible	9 (33.4%)
**Gender**		**Tooth location in the arch**	
Female	20 (74%)	Anterior	18 (66.6%)
Male	7 (26%)	Posterior	9 (33.4%)
**Symptoms**		**Diameter**	
Presence	15 (55.5%)	Small	9 (33.4%)
Absence	12 (44.5%)	Large	18 (66.6%)
**Sinus Tract**		**Endodontic treatment**	
Presence	10 (37.1%)	≤ 4 years	15 (55.5%)
Absence	17 (63.9%)	> 4 years	12 (44.5%)

**Table 2:** Expression of pro-inflammatory and anti-inflammatory cytokines in post-treatment apical periodontitis

Staining intensity	IL-1β	IL-6	TNF-α	IFN-γ	IL-4	TGF-β
**Granuloma**						
Focal	70%	85%	90%	75%	15%	70%
Weak/moderate	25%	15%	10%	25%	80%	10%
Strong	5%	-	-	-	5%	20%
**Cystic epithelium**						
Focal	57%	100%	43%	85.5%	14%	57%
Weak/moderate	28.5%	-	57%	14.5%	57%	43%
Strong	14.5%	-	-	-	29%	-
**Connective tissue of cysts**						
Focal	71%	86%	43%	57%	28.5%	71%
Weak/moderate	-	14%	57%	43%	57%	14.5%
Strong	29%	-	-	-	14.5%	14.5%

**Figure 2:**
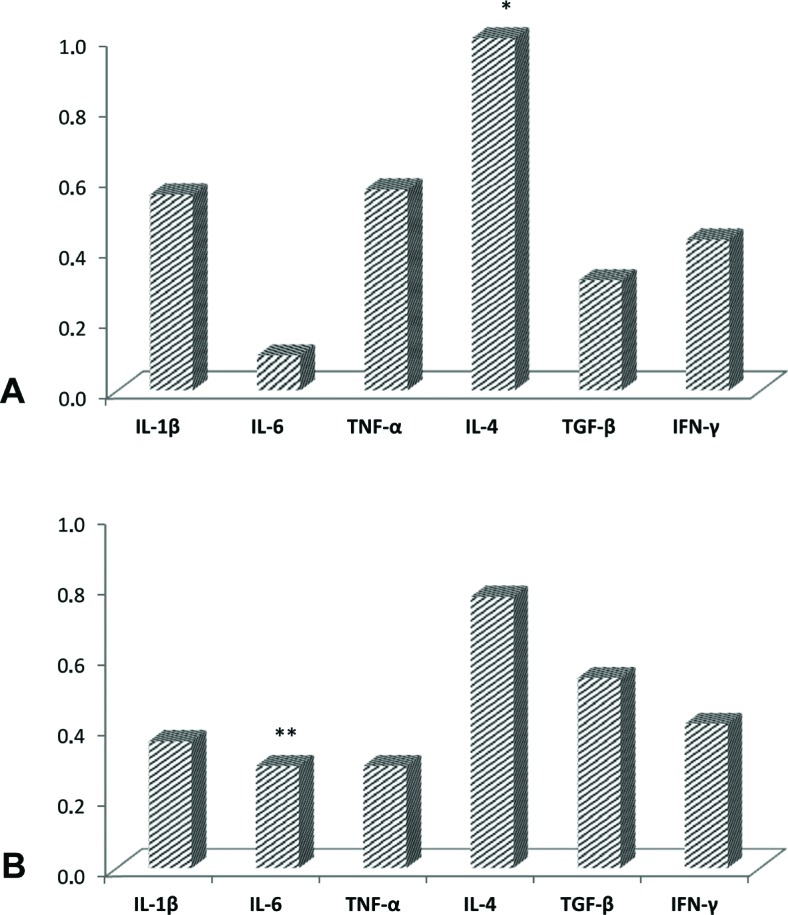
Multiple comparisons between pro-inflammatory and anti-inflammatory cytokines in apical periodontitis lesions. A, granuloma (* p<0.008: IL-4 x IL-6 and IL-4 x TNF-α); B, cyst (** p<0.008: IL-6 x IL-4 and IL-6 x IFN-γ). Wilcoxon multiple comparisons test with Bonferroni correction to p<0.008

Comparison between the pro- and anti-inflammatory cytokines in granulomas revealed that IL-4 expression was significantly higher when compared with IL-6 (p=0.001) and TNF-α (p=0.001) (Figure 2A). Evaluation of the cytokine expression in granulomas associated with different clinical and CBCT data showed a significant relationship only between high levels of TNF-α and small <5 mm AP lesions (p=0.017) (Table 3). There were no statistically significant differences for all other comparisons involving the cytokine expression levels and their relationship with clinical/CBCT features (p>0.05).

**Table 3:** Comparison between pro-inflammatory and anti-inflammatory cytokine expression and clinical and imaging data in periapical granulomas

Clinical/Imaging Data	N	Pro-Inflammatory Cytokines				Anti-Inflammatory Cytokines	
		IL-1β	IL-6	TNF-α	IFN-γ	IL-4	TGF-β
**Symptoms**							
Presence	12	0.48±0.52	0.30±0.10	0.26±0.17	0.43±0.22	0.68±0.34	0.58±0.72
Absence	8	0.17±0.22	0.27±0.28	0.32±0.31	0.37±0.19	0.90±0.26	0.47±0.62
**Sinus Tract**							
Presence	6	0.56±0.61	0.33±0.24	0.23±0.19	0.40±0.17	0.90±0.30	0.56±0.59
Absence	14	0.27±0.34	0.27±0.16	0.31±0.25	0.41±0.22	0.71±0.33	0.52±0.71
**Endodontic treatment**							
≤4 years	12	0.45±0.52	0.33±0.19	0.25±0.15	0.38±0.13	0.80±0.22	0.46±0.68
>4 years	8	0.22±0.27	0.22±0.16	0.35±0.33	0.45±0.29	0.72±0.45	0.65±0.66
**Diameter**							
<5 mm	9	0.28±0.40	0.33±0.20	0.42±0.27 *	0.40±0.14	0.66±0.41	0.55±0.66
≥5 mm	11	0.41±0.49	0.25±0.18	0.18±0.14	0.41±0.26	0.85±0.22	0.52±0.70

Values are expressed as mean ± standard deviation of the scores given for staining intensity.

* p<0.05, Mann-Whitney Test

Comparison between pro-inflammatory and anti-inflammatory cytokines in cysts revealed that IL-6 expression was significant lower than IL-4 (p=0.001) and IFN-γ (p=0.004) (Figure 2B). Evaluation of cytokine expression in cysts associated with different clinical and imaging data disclosed a significant relationship only between high levels of TGF-β expression and cases treated less than 4 years before (p=0.045) (Table 4). All the other comparisons in cyst lesions showed no statistical significance (p>0.05). There was no comparison between the diameter of the lesions and cytokine expression levels because all cysts were classified as large.

**Table 4:** Comparison between pro-inflammatory and anti-inflammatory cytokine expression and clinical and imaging data in periapical cysts

Clinical/Imaging data	N	Pro-inflammatory cytokines				Anti-inflammatory cytokines	
		IL-1β	IL-6	TNF-α	IFN-γ	IL-4	TGF-β
**Symptoms**							
Presence	3	0.66±0.45	0.33±0.08	0.43±0.46	0.43±0.40	1.10±0.53	0.40±0.53
Absence	4	0.47±0.60	0.15±0.29	0.67±0.45	0.42±0.43	0.92±0.55	0.25±0.38
**Sinus Tract**							
Presence	4	0.47±0.60	0.15±0.29	0.67±0.45	0.42±0.43	0.92±0.55	0.25±0.38
Absence	3	0.66±0.45	0.33±0.08	0.43±0.46	0.43±0.40	1.10±0.53	0.40±0.53
**Endodontic treatment**							
≤4 years	3	0.83±0.51	0	0.76±0.46	0.43±0.40	1.03±0.52	0.53±0.48*
>4 years	4	0.35±0.47	0.17±0.29	0.42±0.42	0.42±0.43	0.97±0.57	0.15±0.35

Values are expressed as mean ± standard deviation of the scores given for staining intensity.

* p<0.05, Mann-Whitney Test.

Epithelial and connective tissues of periapical cysts were evaluated

## Discussion

AP is a disease characterized by the accumulation of members of both innate and adaptive immune mechanisms in the periradicular tissues in response to root canal infection^20^. This study used an immunohistochemistry approach to detect the expression of important pro-inflammatory and anti-inflammatory cytokines in human post-treatment AP lesions. Overall, our findings revealed that these categories of cytokines co-existed in the lesions. In addition, the expression levels of some cytokines were related to clinical and imaging manifestations of AP.

Pro-inflammatory mechanisms must be tightly counter-regulated to prevent excessive tissue destruction^23^. The balance between pro-inflammatory and anti-inflammatory cytokines controls the extent of host responses to antigen stimulation within chronic inflammatory processes. In AP, pro-inflammatory cytokines are mostly produced by T_H_1 cells, macrophages and neutrophils, and are involved in the lesion expansion phases because of bone destruction. In contrast, anti-inflammatory cytokines, mostly released by T_H_2 and Treg cells, play an important role in the healing process and restriction of the immune response^15^.

Most cytokines tested in this study exhibited focal expression levels (1 to 5% of the cells stained). This may be justified by the nature of the samples used. The chronic inflammatory AP lesions were associated with teeth in which endodontic treatment or retreatment had been performed at least one year previously. Persistent AP lesions are usually associated with an intraradicular microbiota composed by fewer bacterial cells and species in comparison with primary intraradicular infections^25^. This may result in diverse antigenic load and type. In addition, given the previous treatment, some areas of the lesion might have been under a reparative process. These factors may contribute to a more stable immune response, characterized by a balance between pro- and anti-inflammatory chemical mediators. This balance is also very important to prevent excessive damage associated with the immune response.

IL-4 had the highest expression levels among the tested cytokines. This cytokine is mainly produced by Th2 and mast cells and is known to stimulate the humoral immune response and inhibit both Th1 pro-inflammatory response and bone resorption^2^ ^,^ ^8^ ^,^ ^19^. IL-4 was not significantly related to any of the clinical and CBCT data, which may be explained by the fact that this cytokine occurred at high levels in most specimens. IL-4 may have an important regulatory function in the inflammatory process of post-treatment AP.

TGF-β mostly occurred in focal and weak/moderate expression levels. This mediator can contribute to tissue repair by inhibiting bone resorption and stimulating collagen synthesis, neovascularization, and fibroblast proliferation^2^. TGF-β can inhibit the production of pro-inflammatory cytokines such as IL-1β, TNF-α, and IL-6 by inflammatory cells isolated from both symptomatic and asymptomatic AP lesions^11^. This study found a correlation between TGF-β expression and cysts and the time of endodontic treatment ≤4 years. Our findings, along with other studies^1^ ^,^ ^9^, suggest a possible role for TFG-β in stabilizing AP by its immunosuppressive and regulatory effects.

IL-1β is an important cytokine involved in the stimulation of bone resorption, accounting for a large percentage of resorptive activity in AP^13^. In this study, the expression levels of IL-1β were usually focal and weak/moderate, which possibly indicates a chronic stable stage for the post-treatment AP inflammatory process. No correlations were observed between IL-1β and clinical and imaging manifestations.

Low expression of TNF-α was observed in both granulomas and cysts. Danin, et al.^9^ (2000) reported lower detection of TNF-α in granulomas, while it was not detectable in samples of cysts. Teixeira-Salum, et al.^27^ (2010) reported statistically higher levels of TNF-α in cysts in comparison with granulomas. This cytokine was significantly more expressed in small granulomas. TNF-α has the capacity to induce bone resorption through the activation of osteoclasts and stimulation of mediators that destroy the extracellular matrix of bone tissue^9^ ^,^ ^11^ ^,^ ^26^. The low expression of this important pro-inflammatory mediator may also be related to the chronic stage of post-treatment disease.

Although it is a pro-inflammatory cytokine, IFN-γ can suppress pathological bone resorption associated with inflammation because it inhibits the activation of mature osteoclasts and bone resorption stimulated by IL-1 and TNF^2^ ^,^ ^19^. Since IFN-γ had low expression in both granulomas and cysts in this study, it is difficult to infer its role in the pathobiology of post-treatment AP.

IL-6 is involved in B-lymphocyte differentiation in plasma cells and stimulation of bone resorption^6^ ^,^ ^12^. It was the cytokine tested with the lowest level of expression even in symptomatic and large AP lesions, which is in disagreement with a previous study^11^. Nevertheless, rats that were genetically incapable of producing IL-6 presented increased bone destruction^6^. This suggests that IL-6 has both pro- and anti-inflammatory functions; the final effect depends on the target-cells and their relationship with other cytokines released in the environment.

This and other studies have looked for associations between cytokine expression and clinical/imaging manifestations of AP. In this study, TNF-α expression levels were significantly associated with small granulomas (<5 mm in diameter); while levels of TGF-β were correlated with cysts and treatments performed <4 years before. No significant associations with clinical and CBCT images were observed for the other cytokines tested. Martinho, et al.^18^ (2012) reported a relationship between lesion size and IL-6, and exudation and TNF-α. IL-6 expression has also been related to symptomatic lesions^5^ ^,^ ^21^. TNF-α expression was not significantly increased in the presence of symptoms^4^ ^,^ ^21^, but it was associated with large lesions^11^, in contrast with our study. IL-1β has also been related to symptoms of AP^11^ ^,^ ^16^. However, Lim, et al.^17^ (1994) found no association between the levels of IL-1β and different clinical characteristics, which is in accordance with our study. Colic, et al. ^8^ (2009) suggested a relationship between symptomatic AP and the presence of neutrophil infiltrate with IL-17 and IFN-γ.

## Conclusion

In conclusion, the present findings revealed a balance between the expression of pro- and anti-inflammatory cytokines associated with chronic inflammatory process in post-treatment AP. Of the target cytokines, TNF-α were associated with small <5 mm AP lesions and TGF-β with cases treated less than 4 years before.
